# Classification of temporal lobe epilepsy based on neuropsychological tests and exploration of its underlying neurobiology

**DOI:** 10.3389/fnhum.2023.1100683

**Published:** 2023-06-14

**Authors:** Xianghong Meng, Kan Deng, Bingsheng Huang, Xiaoyi Lin, Yingtong Wu, Wei Tao, Chuxuan Lin, Yang Yang, Fuyong Chen

**Affiliations:** ^1^Department of Neurosurgery, Shenzhen University General Hospital, Shenzhen, China; ^2^Medical AI Lab, School of Biomedical Engineering, Health Science Center, Shenzhen University, Shenzhen, China; ^3^MSC Clinical and Technical Solutions, Philips Healthcare, Guangzhou, China; ^4^Department of Radiology, Suining Central Hospital, Suining, China

**Keywords:** temporal lobe epilepsy, machine learning, structural magnetic resonance imaging, neuropsychology, cognitive impairment

## Abstract

**Objective:**

To assist improving long-term postoperative seizure-free rate, we aimed to use machine learning algorithms based on neuropsychological data to differentiate temporal lobe epilepsy (TLE) from extratemporal lobe epilepsy (extraTLE), as well as explore the relationship between magnetic resonance imaging (MRI) and neuropsychological tests.

**Methods:**

Twenty-three patients with TLE and 23 patients with extraTLE underwent neuropsychological tests and MRI scans before surgery. The least absolute shrinkage and selection operator were firstly employed for feature selection, and a machine learning approach with neuropsychological tests was employed to classify TLE using leave-one-out cross-validation. A generalized linear model was used to analyze the relationship between brain alterations and neuropsychological tests.

**Results:**

We found that logistic regression with the selected neuropsychological tests generated classification accuracies of 87.0%, with an area under the receiver operating characteristic curve (AUC) of 0.89. Three neuropsychological tests were acquired as significant neuropsychological signatures for the diagnosis of TLE. We also found that the Right-Left Orientation Test difference was related to the superior temporal and the banks of the superior temporal sulcus (bankssts). The Conditional Association Learning Test (CALT) was associated with the cortical thickness difference in the lateral orbitofrontal area between the two groups, and the Component Verbal Fluency Test was associated with the cortical thickness difference in the lateral occipital cortex between the two groups.

**Conclusion:**

These results showed that machine learning-based classification with the selected neuropsychological data can successfully classify TLE with high accuracy compared to previous studies, which could provide kind of warning sign for surgery candidate of TLE patients. In addition, understanding the mechanism of cognitive behavior by neuroimaging information could assist doctors in the presurgical evaluation of TLE.

## 1. Introduction

Nowadays, temporal lobe epilepsy (TLE) remains the most common type of epilepsy, with an incidence of more than 50% among patients with epilepsy. TLE is also the most common form of drug-resistant epilepsy, and about 70% of patients are drug-resistant (Abarrategui et al., 2021). Typical anterior temporal lobectomy (ATL) is the most effective treatment to control seizure, about 63.8% will have seizure freedom after surgery (Xu et al., 2020). However, even after complete removal of the anterior temporal lobe and mesial structures, a significant number of patients will experience seizures again which suggests that some epileptogenic tissue has not been resected. It is possibly due to pseudotemporal epilepsy whereby a extratemporal epileptogenic zone (extraTLE) was misdiagnosed, dual pathology combining an extratemporal epileptogenic lesion and temporal plus epilepsy (TPE), which could mimic the semiology and electroencephalographic (EEG) patterns including interictal and ictal features (Eryurt et al., 2015; Suresh et al., 2015; Barba et al., 2016). The most frequent ictal presentations of TLE include loss of responsiveness preceded by aura, oral automatism, ipsilateral gestural automatisms and contralateral limb dystonia or immobility. Abdominal and psychic auras are most common, which are not always pathognomonic of a temporal origin. They can also be elicited by the other regions of limbic system including insula, orbitofrontal region, anterior cingulum. And interictal and ictal scalp EEG could not distinguish them, especially to TLE and TPE, which make more complicated for accurate localization (Barba et al., 2016; Abarrategui et al., 2021). Distinguishing TLE from extraTLE and TPE has important clinical significance for surgical outcome before performing anterior temporal lobectomy (Frank et al., 2018). To determine who would benefit from ATL, presurgical evaluation of TLE is usually performed using high-resolution magnetic resonance imaging (MRI) due to its high sensitivity and specificity for hippocampal sclerosis (HS), the most common pathologic basis of TLE. However, seizure-free rates for those with normal MRI results about 50% (Muhlhofer et al., 2017). Therefore, it is necessary to propose a new method to assist clinicians in the identification of TLE patients whose disabling seizures could be resolved by standard anterior temporal lobectomy, improve long-term postoperative seizure-free rates, and improve patients’ quality of life, especially for MRI negative patients. Or there will be signs that could send red flag to the non-TLE patients who are misdiagnosed as TLE undergoing ATL procedure.

Neuropsychological assessment is an essential diagnostic tool for evaluating human brain function. Before the advent of MRI, clinicians used the patterns of cognitive strengths and weaknesses derived from a neuropsychological assessment to lateralize and localize cerebral pathology. The difficulties on particular neuropsychological tasks represent dysfuncion within the underlying network that subserves this function, which could be related to many factors. Epilepsy is considered as the disorder of cerebral networks. It is hard to assigning one cognitive dysfunction to a certain brain structure. Vice versa, one focal lesion could related to multiple cognitive dysfunctions. Cognitive impairment is a common comorbidity in patients with TLE. Moreover, memory decline is a major cognitive impairment in patients with TLE. However, most studies have reported the TLE have the involvement of other cognitive abilities, including attention, language, praxis, executive function, judgment, insight, and problem solving (Hermann et al., 2006; Keary et al., 2007; Allone et al., 2017). Besides, currently the interpretation of relies immensely on clinicians’ experience. They need to take into account the results of multiple neuropsychological assessment, the influence factors such as education, gender and intelligence, which is complicated, subjectives and time-consuming. Therefore, it is difficult to draw a conclusion through neuropsychological tests alone as patients with epilepsy often have multiple cognitive impairments (Hermann et al., 1997; Martin et al., 2000; Sung et al., 2013; Hamberger, 2015). State-of-the-art analysis techniques, such as machine learning, may be more objective and helpful for neuropsychological analysis. Studies have employed support vector machine (SVM) and neuropsychological tests to classify TLE and extraTLE with an accuracy of 78% (Frank et al., 2018). Another study reported 74% accuracy when distinguishing TLE from the healthy control group, which was distinguished using SVM with 14 neuropsychological tests (Hwang et al., 2019). These studies indicated that using machine learning methods with neuropsychology tests can effectively assist clinicians in preoperative evaluation. However, this technique has not been well developed in epilepsy research. Moreover, different tests have different effects on the classification results. Therefore, it is necessary to explore which tests are most effective for TLE classification.

Understanding the brain structural mechanism of cognitive behavioral changes can provide a theoretical basis for locating the epileptogenic zone in presurgical evaluation. Some studies have explored the relationship between cognitive impairment and abnormal brain structure in patients with TLE. Studies have shown that abnormalities in gyrification are associated with a significantly lower performance intelligence quotient (IQ), poorer verbal and visual memory, significant slowing across measures of simple and complex psychomotor processing as well as the speed of fine motor dexterity (Oyegbile et al., 2004). Decreased volume of cerebral gray matter is significantly associated with a lower IQ, as well as a decline in immediate and delayed memory, executive function, and speed of motor dexterity (Oyegbile et al., 2006). The lower IQ is a typical symptom of TLE. Although studies have investigated the changing patterns of cortical surface in patients with TLE, the mechanism underlying abnormal cognitive behavior remains unclear.

This study aimed to identify which neuropsychological tests may be effective for distinguishing TLE from extraTLE and explore the structural mechanism underlying abnormal cognitive behavior using MRI. We hypothesized that neuropsychological test could distinguish TLE from extraTLE. Furthermore, we expected that the brain differences between TLE and extraTLE would be associated with the selected neuropsychological test and can provide a theoretical basis for TLE classification.

## 2. Materials and methods

### 2.1. Data acquisition

Patients diagnosed with drug-resistant focal epilepsy who were either had seizure-related lesion or underwent stereoelectroencephalogram (SEEG) in the epilepsy centers of Shenzhen University General Hospital and Shenzhen secondary people’s Hospital from 2016 to 2019, were selected by retrospective review. They initially underwent phase one non-invasive pre-surgical evaluation that included patient and family history followed by video electroencephalography and neuropsychological testing. Consequently, each patient underwent 3T magnetic resonance imaging (MRI) and functional imaging (inter-ictal FDG-PET scan). Invasive exploration using SEEG was considered necessary to delineate the epileptogenic zone, map the functional cortex and to determine the limits of the resection. All patients underwent surgery and had postsurgical outcome consistent with Engel I or II criteria. Patients were included if they (1) were right-handed; (2) were aged ≥ 18 years; (3) can complete the neuropsychological tests independently, and attained a normal level on the Wechsler Adult Intelligence Scale-Revised Chinese version (WAIS-RC); (4) followed-up at least for 1 year after surgery. Patients were excluded if they had any of the following: multifocal epilepsy; medical illness with central nervous system impact other than epilepsy; drug abuse; or serious organ diseases, such as brain tumor, intracranial hemorrhage, and metabolic diseases. Twenty-three patients were classified as TLE by experienced neurologists and neurosurgeons based on the International League Against Epilepsy diagnosis criteria (Fisher et al., 2017; Scheffer et al., 2017), SEEG results, surgical plan and postsurgical outcome. Mesial temporal lobe epilepsy and the neocortical epilepsy over anterior temporal lobe were included in the TLE group, whose resection regions were located within the areas of standard temporal lobectomy. Similarly, there were 23 patients in the extraTLE group. The extraTLE group included patients with frontal lobe, parietal lobe, and occipital lobe epilepsy. The temporal plus epilepsy patients were defined based on SEEG results, whose primary temporal lobe epileptogenic zone extending to the insula, the suprasylvian operculum, the orbito-frontal cortex and the temporo-parieto-occipital junction, were also included in the extraTLE group. The reason of this grouping criterion is to provide warning sign for forecasting the poor postsurgical outcome of standard temporal lobectomy.

We collected detailed information, including demographic data, history, seizure semiology, long-term scalp video-EEG monitoring, neuropsychological tests, and neuroimaging findings. The Ethics Committee at the Department of Neurosurgery, Shenzhen University General Hospital, approved this study, and all participants provided written informed consent.

A comprehensive neuropsychological evaluation explores several cognitive domains, mainly including perception, memory, attention, executive function, language, motor and visuomotor function (Baxendale et al., 2019), which were included in our neuropsychological testing battery. Neuropsychological testing could provide important information about the cognitive conditions of epilepsy patients, but it could be influenced by multiple factors, such as antiseizure medications, pre-testing seizures, psychiatric comorbidity (e.g., depression) and sleep (Baxendale et al., 2019). All patients underwent neuropsychological tests at their first visit to the hospital. We controlled the influence factors by choosing the time without pre-testing seizures, good sleep before testing and stopping topiramate for at least 3 days before testing. Wechsler Adult Intelligence Scale-Revised Chinese version (WAIS-RC), revised by Gong (1983), is the most popular intelligence scale used in China. Five subtests were choosed to roughly evaluate the general cognitive function, including Similarity Test, Arithmetic Test, Digital Span Test (forward and backward), Block-design Test and Object Assembly. The subtest score was converted to a standardized score based on age and living background (Urban vs. Rural). The standardized score of verbal IQ was calculated by the average of standardized score of Similarity, Arithmetic and Digital span and the standardized score of performance IQ was calculated by the average of standardized score of Block-design and Object Assembly. The average standardized IQ score is above 9 was considered as normal. Memory ability was assessed using the Abstract Figures Learning Test (AFLT), Abstract Verbal Learning Test (AVLT) (Majdan et al., 1996), Real Auditory Verbal Learning Test (RAVLT), and the Batterie d’Efficience Mnésique (BEM) Test. Visuoperceptual skills were assessed using the Face Recognition Test, Line Orientation Test, Right-Left Orientation Test, and Rey Complex Figure Test. Executive functions were assessed using the Self-Ordered Pointing Test (SOP) (Ross et al., 2007), Selective Attention Test, Stroop Test, Conditional Association Learning Test (CALT) (Petrides, 1985, 1997), Figure Fluency Test, Category Verbal Fluency Test, Phonic Verbal Fluency Test, and Component Verbal Fluency Test. The Token Test (De Renzi and Vignolo, 1962) and Boston Naming Test (Tombaugh and Hubiey, 1997) were administered to assess language functions. All neuropsychological tests are shown in the Supplementary Table 1.

Magnetic resonance imaging scans of 46 patients were performed on three different scanners. A total of 39 patients were scanned with the SIEMENS 3.0 Tesla MR scanners, 2 patients with the uMR790 3.0 Tesla MR scanner, and 5 patients with the GE 3.0 Tesla MR scanner. The detailed scanning parameters are listed in Table 1.

**TABLE 1:** Magnetic resonance imaging (MRI) acquisition parameters.

Instrument manufacture	Slices	Slice thickness (mm)	TR, TE (ms)
SIEMENS	176	1	1900, 2.9/2530, 3.0
	192	1	5000, 3.0
	176	1.2	2200, 1.5
GE	352/336	1	8200, 3.2
uMR790	176/160	1	7200, 3.1

TR, repetition time; TE, echo time.

### 2.2. Classification of TLE and extraTLE with neuropsychological tests

Twenty-three neuropsychological tests were included in this study, and the neuropsychological test data were normalized using min-max normalization. All neuropsychological tests may contain some redundant or highly relevant features that affected the performance of the model. Herein, we used the least absolute shrinkage and selection operator (LASSO) algorithm for feature selection. Feature selection was achieved by adding L1 penalty to the objective function, which caused many coefficients to be close to zero, with only a small subset to be non-zero. LASSO regression can better deal with multicollinearity (Oyeyemi et al., 2015).

A logistic regression algorithm was employed for TLE classification using scikit-learn toolkit.^1^ The classification performance was evaluated using leave-one-out cross-validation with N (N = total number of instances) iterations. At each iteration, a single subset was retained as the validation data for test the model, and the remaining N-1 subsets were used as the training data. In the first step, the LASSO algorithm was applied to the training data for feature selection. The neuropsychology tests that occurred most times in the training data at all iterations were considered as the significant neurologic signatures for TLE classification. Thereafter, the selected features were input into the logistic regression classifiers to predict the classification outcome using leave-one-out cross-validation.

The performance of the classification models was evaluated using the receiver operating characteristic curve (ROC) and the area under the ROC curve (AUC) was calculated. We also evaluated the models by evaluating the accuracy, specificity, and sensitivity, which were calculated as follows:


Sensitivity=TP/(TP+FN)

Specificity=TN/(TN+FP)

Accuracy=(TP+TN)/(TP+FP+FN+TN)

where, TP, TN, FP, and FN denote true positive, true negative, false positive, and false negative, respectively, calculated according to the optimal cut-off value that maximized the Youden index.

### 2.3. Correlations of neuroimaging and neuropsychological tests

It is no doubt that analysis of the pattern of cognitive behavior change could provide the clue for lateralization and localization of the epileptogenic zone especially for the MRI negative patients. Overall assessment covering different cognitive domain could give us some information, but it still need experienced neuropsychologists in epilepsy. By exploring the underlying the relationship between structural alterations with the cognitive abnormalities, it might not only help us to understand the underlying mechanism of behavior change, but also could backstep the location of possible lesion or dysfunction of certain brain network.

Images were preprocessed and analyzed using FreeSurfer (version stable v6.0.0).^2^ The image preprocessing includes skull stripping, volumetric labeling, intensity normalization, white matter segmentation, surface atlas registration, surface extraction, and gyral labeling. To improve the signal-to-noise ratio, a 10-mm full width at half-maximum was used for smoothing. Cortical thickness, sulcal depth, mean curvature, gray-white matter contrast, and gray matter volume were computed after image preprocessing and reconstruction. Cortical thickness was measured as the shortest distance at the corresponding vertex between the white matter surface and the pial surface (Fischl and Dale, 2000). The sulcal depth is defined as the geodesic from the vertex located within the sulci to the closest vertex within the gyral crown (Fischl et al., 1999). The mean curvature is the reciprocal of the radius of the inscribed circle measured at the gray-white matter boundary, which is equal to the average of the main curvatures k1 and k2 (Pienaar et al., 2008). The gray-white matter contrast is calculated as the ratio of gray matter signal intensity to white matter signal intensity (Salat et al., 2009). Gray matter volume is defined as the amount of gray matter that lies between the gray-white interface and the pia matter (Winkler et al., 2010).

A generalized linear model was used to explore the relationship between the MRI features and the selected neuropsychological test, and then the coefficient that differed between TLE and extraTLE were compared. Statistical maps were generated using FreeSurfer’s Query, Design, Estimate, Contrast interface tools, accounting for the effects of the covariates of sex and age. If two or more neuropsychological tests that evaluate the same cognitive function were selected, one neuropsychological test was considered as an independent variable, whereas the others were considered as nuisance for generalized linear model analysis. Multiple comparisons were corrected with a Monte Carlo simulation using a *p*-value < 0.05. The results were visualized by overlaying significant areas onto the inflated cortical surface.

### 2.4. Statistical analysis

Statistical analyses were performed using Statistical Package for the Social Sciences (SPSS) Version 20. Demographic data (i.e., age, sex, background, education, age at onset, and IQ) were compared between the groups with TLE and extraTLE using Chi-square test for categorical variables, two-sample *t*-test for continuous variables, if they had a normal distribution, or using Mann–Whitney U test for continuous variables with non-normal distribution. Statistical significance was defined as a *p*-value < 0.05.

## 3. Results

### 3.1. Participant demographic characteristics

There were ninety-nine patients with refractory epilepsy had finished evaluation with full datas of MRI and neuropsychological testing in Shenzhen University General Hospital and Shenzhen Second People Hospital from 2016 to 2019. Fifty-five patients have done epileptogenic zone resection and their outcome are consistent with Engel Ia and Ib. Among them, nine patients were excluded due to IQ below normal range. Twenty-three patients with TLE (ages 19–51 years, mean age: 33.6 ± 2.2 years) and 23 patients with extraTLE (ages 18–55 years, mean age: 30.7 ± 1.8 years) met the criteria for inclusion in this study. The extraTLE group included patients with 12 frontal lobe epilepsy(52.2%), 6 temporal plus temporo-parieto-occipital (TPO) junction epilepsy (26.1%), 1 parietal lobe epilepsy (4.3%), 2 insular & operculum epilepsy (8.7%) and 2 others (ventricular heterotopia and hypothalamus hamartoma epilepsy, 8.7%). Participant demographic characteristics are shown in Table 2. There were no differences in age, sex, background, age at onset, or education between groups. The TLE group showed higher IQ than the extraTLE group, and the difference was statistically significant (*p* < 0.001).

**TABLE 2:** Patient demographics characteristics.

Characteristics	TLE (*n* = 23)	ExtraTLE (*n* = 23)	*p*-value^a^
Age, mean ± SD, years	33.6 ± 2.2	30.7 ± 1.8	0.422
Sex			0.234
Men	11	15	
Women	12	8	
Background			0.536
Urban	9	7	
Rural	14	16	
Age at onset, mean ± SD, years	20.7 ± 2.6	15.3 ± 1.5	0.079
Education, mean ± SD, years	11.9 ± 0.6	10.3 ± 0.8	0.136
IQ, mean ± SD	12.6 ± 0.3	10.5 ± 0.4	<0.001

TLE, temporal lobe epilepsy; extraTLE, extratemporal lobe epilepsy; IQ, intelligence quotient; SD, standard deviation.

^a^Comparison between the TLE group and the extraTLE group.

### 3.2. Classification of TLE and extraTLE with neuropsychological tests

The neuropsychology tests of the Right-Left Orientation Test and Component Verbal Fluency Test occurred 46 times in the training data sets, and the CALT occurred 45 times in the training data sets, while other neuropsychological tests occurred less than twice in the training data sets. Hence, three neuropsychological tests of the Right-Left Orientation Test, CALT, and Component Verbal Fluency Test were finally selected as significant neuropsychological signatures for the diagnosis of TLE.

**FIGURE 1:**
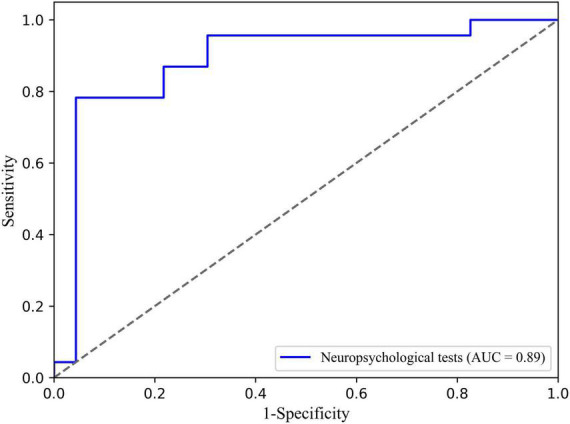
Receiver operating characteristic (ROC) curve of the TLE classification based on neuropsychological tests and logistic.

The logistic regression classification based on selected neuropsychological tests in distinguishing TLE from extraTLE was achieved with an average accuracy of 87.0%, sensitivity of 95.7%, specificity of 78.3%, and AUC of 0.89. The ROC curve is shown in Figure 1.

### 3.3. Correlations of neuroimaging and neuropsychological tests

**FIGURE 2:**
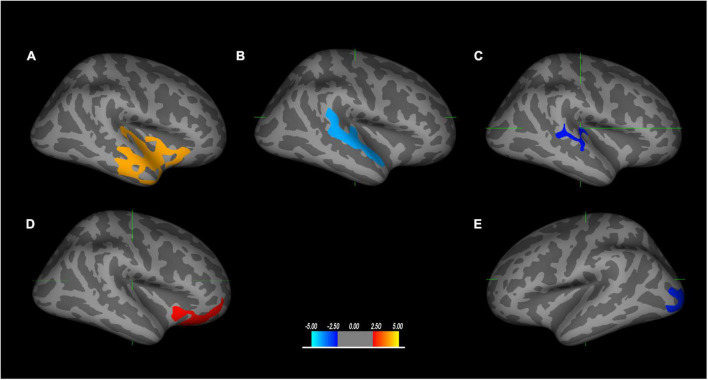
The brain regions correlated with neuropsychological tests are significantly different between TLE and extraTLE, accounting for age and sex. The relationship between the Right-Left Orientation Test and the gray matter volume difference in the superior temporal area **(A)**, the sulcal depth difference in the banks of the superior temporal sulcus (bankssts) **(B)**, and the mean curvature difference in the bankssts area **(C)**, respectively. The relationship between the Conditional Association Learning Test (CALT) and the cortical thickness difference in the lateral orbitofrontal **(D)**. The relationship between the Component Verbal Fluency Test and the cortical thickness difference in the occipital area **(E)**. The values in the color bar represent log10 (*p*-value) (*p* < 0.05).

The correlations between structural abnormalities and cognitive impairment are presented in Figure 2 and Table 3. The Right-Left Orientation Test difference was related to the difference in gray matter volume in the superior temporal region, the difference in sulcal depth in the banks of the superior temporal sulcus (bankssts), and the mean curvature difference in bankssts between TLE and extraTLE, while accounting for sex, age and total intracranial volume. CALT was associated with the cortical thickness difference in the lateral orbitofrontal area between the two groups. The Component Verbal Fluency Test was associated with the cortical thickness difference in the occipital pole between the two groups.

**TABLE 3:** Correlations between the brain region and the neuropsychological tests.

Cognitive function	Neuropsychology	Brain regions associated with neuropsychological tests						
		**Hemisphere**	**Brain region**	**Talairach coordinates**			**Lobes**	**Cortical surface features**
				**TalX**	**TalY**	**TalZ**		
Visuoperceptual skill	Right-Left Orientation Test	R	Superior temporal	49.2	−0.2	−6.8	Temporal	Gray matter volume
		R	Bankssts	54.8	−31.9	4.9	Temporal	Sulcal depth
		R	Bankssts	49.4	−33.6	6.4	Temporal	Mean curvature
Executive functions	Conditional Association Learning Test (CALT)	R	Lateral orbitofrontal	34.4	42.5	−10.3	Frontal	Cortical thickness
	Component Fluency Test	L	Lateral occipital	−23.0	−90.2	6.0	Occipital	Cortical thickness

## 4. Discussion

In this study, the Right-Left Orientation Test, CALT, and Component Verbal Fluency Test were finally selected as significant neuropsychological signatures for the diagnosis of TLE according to the analysis of the LASSO algorithm. An 87% accuracy was achieved when using logistic regression with selected neuropsychological tests for TLE classification. Similar research has shown that 78% accuracy was obtained when using multiple neuropsychological assessment and SVM methods to distinguish the TLE from the extraTLE (Frank et al., 2018). The application of machine learning approaches with the neuropsychological test for epilepsy classification is extremely uncommon, but these studies have verified the feasibility and validity of the method.

Among these selected neuropsychological tests, the Right-Left Orientation Test was used to assess visuospatial perceptual skills. Traditional models confirmed that the visual pathway is segregated into two distinct streams: the temporal-occipital ventral pathway and the occipital-parietal dorsal pathway (Sheth and Young, 2016). The dorsal pathway mainly processes information about object location, developing relations to several areas of the cortex, such as the frontal, temporal, and limbic lobes (Trés and Brucki, 2014). Our results showed that the Right-Left Orientation Test difference was related to the difference in gray matter volume in the anterior to posterior superior temporal region (including temporoparietal junction), the sulcal depth difference in bankssts, and the mean curvature difference in the bankssts; the coefficient is significantly different between TLE and extraTLE, which may indicate that this region plays a key role in the behavior difference between the two groups in Right-Left Orientation Test. Left-right disorientation was one symptom of Gerstmann syndrome, and was considered as the dysfunction of dominant parietal lobe, especially in angular gyrus. However, insular and operculum lesion also could cause Gerstmann syndrome, resulting in a pseudoparietal presentation (Bhattacharyya et al., 2014). Superior temporal gyrus, neighboring to angular gyrus, have close connectivity with insular and parietal lobe, which may influence the capacity of left-right orientation. Due to the limited number of the patients with parietal lobe epilepsy in the extraTLE group, it may be the reason why we did not obtain the significant difference in gray matter volume of parietal cortex between the two groups.

The CALT and the Component Verbal Fluency Test were used to assess executive function skills. More specifically, the parts of the brain that deal with memory and conditional associative learning are activated during the CALT. Our results showed that CALT was associated with the cortical thickness difference in the lateral orbitofrontal cortex, and that the coefficient was significantly different between TLE and extraTLE. Conditional associative learning can facilitate memory processing. Consequently, repeated memory is also an important part of conditional response formation. The lateral orbitofrontal is the main brain area connecting with them. Previous reports have indicated that the orbitofrontal cortex is strongly connected with the limbic area of the medial temporal lobe, which is closely related to the establishment of declarative memory (Petrides, 2007; Barbey et al., 2011). OFC neuronal activity represents a long-term associative memory to support behavioral adaptation (Namboodiri et al., 2019). Orbitofrontal damage may impair the exclusionary aspect of attention. Our result is also consistent with previous findings that executive function is associated with the frontal lobe (Della Sala et al., 1998; Schnider et al., 2020), and these findings may indicate that these two groups have different degrees of correlation in the frontal lobe, which may be the reason that the CALT can distinguish TLE from the extraTLE. Previous studies have shown that the frontal lobe plays an important role in executive function, which is an extensive efferent and afferent connection to most brain regions (Heyder et al., 2004). Cortico-subcortical circuits that connect the prefrontal cortex, the basal ganglia, and the cerebellum via the thalamus are considered as the neuroanatomical basis for executive function (Bostan et al., 2010). Therefore, frontal impairment may cause damage to other brain regions associated with executive dysfunction. The integrity of the frontal cortex is necessary but not sufficient for intact executive function (Della Sala et al., 1998). Executive dysfunction may not always reflect frontal lesions, but probably indicate damaged neural networks throughout the brain. Fluency was defined as the ability to maximize unique response productions, and at the same time to avoid or minimize response repetition, including verbal and non-verbal categories (Ruff et al., 1994). It has been suggested that all forms of fluency, independent from modality, represent general executive functions (Zalonis et al., 2017). Non-verbal fluency is related not only to executive function but also to visuomotor abilities (Ruff et al., 1987). Right-posterior parietal cortex and left temporal cortex are more especially involved in figural and semantic fluency (Ghanavati et al., 2019). Chinese is one kind of non-alphabetic language, originating from hieroglyphs with dual features of verbal and graphic, and may involve diffuse brain region, especially in frontal and temporal lobe. This may be the reason that Component Verbal Fluency Test was associated with the cortical thickness difference in the occipital pole region. This also proposed a supermodal contribution to the executive function, which was consistent with the previous study (Zalonis et al., 2017). Occipital lobe has very close connectivity with the frontal lobe by frontooccipital fasciculus. The dysfunction of occipital lobe may also can cause the decline of visual executive function.

A few limitations of this study should be noted. First, the small sample size of patients with epilepsy may result in decreased power to distinguish TLE from extraTLE using a machine learning algorithm. Models developed based on such a small sample size may not yet be adequate for the clinical diagnosis of TLE. The relatively small sample size may also affect the analysis of the correlations between structural abnormalities and cognitive impairment. Second, we only used structural MRI, but not functional MRI, DTI, or other modalities to analyze the correlations between the structural abnormalities and the cognitive impairment, which may not be comprehensive. Most studies have demonstrated that TLE not only shows hippocampal atrophy and signal change on magnetic resonance images, but also shows functional connectivity abnormalities (Miró et al., 2015; Bharath et al., 2019). We did not analyze from the perspective of functional connectivity. Third, the MRI scanning parameters were significantly different between individuals, which may lead to the deviation in preprocessing and can further influence the result. Future studies with a larger sample size of patients should continue to investigate the mechanism of cognitive impairment in patients with TLE and to build a more reliable machine learning model for TLE classification. Moreover, use of additional multimodalities may be necessary for in future studies in patients with TLE. From clinical observation, frontal vs. temporal lobe epilepsy patients could combine different psychiatric comorbidity, depression and anxiety more popular in temporal lobe epilepsy and schizophrenia with higher occurrence in frontal lobe epilepsy. Psychiatric evaluation as well as more sensitive neuropsychological test, like Logical Memory and Recognition Test (discriminating frontal vs. temporal), could be added to the testing battery in future (Paradiso et al., 2001; Conradi et al., 2020).

## 5. Conclusion

Our work advocated that using a machine learning model with neuropsychological data can successfully classify TLE with high accuracy. Furthermore, we explored significant neurologic signatures for TLE classification, providing a new path for the diagnosis of TLE. Our study send a potential red flag for ATL when the patients had abnormal performance of Right-Left Orientation Test, CALT and Component Verbal Fluency Test. The findings about the relationship between structural abnormalities and cognitive impairment can better assist physicians in the preoperative assessment, surgical planning, surgical outcome prediction, and surgical rehabilitation planning for patients with TLE.

## Data availability statement

The original contributions presented in this study are included in the article/Supplementary material, further inquiries can be directed to the corresponding authors.

## Ethics statement

The studies involving human participants were reviewed and approved by the Department of Neurosurgery, Shenzhen University General Hospital, Shenzhen, China. The patients/participants provided their written informed consent to participate in this study.

## Author contributions

FC, XM, and YY conceived and designed the experiment. FC provided the administrative support. XM collected and delineated the images and collected the clinical data on patients. KD performed the experimental design, analyzed the data, and prepared the graphs. XM and KD wrote the manuscript. BH, XL, YW, WT, and CL assisted with the manuscript writing and data analysis. All authors read and approved the final version of the manuscript.

## Footnotes

1
https://scikit-learn.org/stable

2
http://surfer.nmr.mgh.harvard.edu
