# Supplementary material for: Classification of temporal lobe epilepsy based on neuropsychological tests and exploration of its underlying neurobiology
Source: Front Hum Neurosci. 2023 Jun 14;17:1100683. doi: 10.3389/fnhum.2023.1100683 (PMC10307531; doi:10.3389/fnhum.2023.1100683)
Supplement: Supplementary file 1 [file Table_1.DOCX]

Supplementary Material

# Supplementary Table S1: **Neuropsychological tests.**

| **Cognitive function** | **Neuropsychological tests** |
| --- | --- |
| **WAIS-RC** |  |
| Verbal IQ |  |
| Comprehension function | Similarity Test |
| Arithmetic function | Arithmetic Test |
| Working memory and attention | Digit Span Test (forward and backward) |
| Performance IQ |  |
| Visumotor function | Object Assembly |
| Visuospatial function | Block-design Test |
| **Memory ability** |  |
| Verbal Memory Ability | Abstract Verbal Learning Test (AVLT)  Real Auditory Verbal Learning Test (RAVLT) |
| Non-Verbal Memory Ability | Abstract Figures Learning Test (AFLT)  The Batterie d'Efficience Mnésique (BEM) Test |
| **Visuoperceptual skills** | Face Recognition Test  Line Orientation Test  Right-Left Orientation Test  Rey Complex Figure Test |
| **Executive functions** | Self-Ordered Pointing Test (SOP)  Selective Attention Test  Stroop Test  Conditional Association Learning Test (CALT)  Figure Fluency Test  Category Verbal Fluency Test  Phonic Verbal Fluency Test  Component Verbal Fluency Test |
| **Language functions** | The Token Test  Boston Naming Test |

Note. WAIS-RC, Wechsler Adult Intelligence Scale-Revised Chinese version; IQ, intelligence quotient; SD, standard deviation.
